# Supplementary material for: Repurposing High-Throughput Screening Identifies Unconventional Drugs with Antibacterial and Antibiofilm Activities against Pseudomonas aeruginosa under Experimental Conditions Relevant to Cystic Fibrosis
Source: Microbiol Spectr. 2023 Jun 12;11(4):e00352-23. doi: 10.1128/spectrum.00352-23 (PMC10433973; doi:10.1128/spectrum.00352-23)
Supplement: Supplemental file 1 — Supplemental material. Download spectrum.00352-23-s0001.docx, DOCX file, 0.09 MB [file spectrum.00352-23-s0001.docx]

**Repurposing high-throughput screening identifies unconventional drugs with antibacterial and antibiofilm activities against *Pseudomonas aeruginosa* under experimental conditions relevant to cystic fibrosis**

Giovanni Di Bonaventura,^a,b#^ Veronica Lupetti,^a,b^ Andrea Di Giulio,^c^ Maurizio Muzzi,^c^ Alessandra Piccirilli,^d^ Lisa Cariani,^e^ Arianna Pompilio^a,b^

^a^ Department of Medical, Oral, and Biotechnological Sciences, “G. d’Annunzio” University of Chieti-Pescara, Chieti, Italy.

^b^ Center for Advanced Studies and Technology, “G. d’Annunzio” University of Chieti-Pescara, Chieti, Italy.

^c^ Department of Science, University Roma Tre, Rome, Italy.

^d^ Department of Biotechnological and Applied Clinical Sciences, University of L'Aquila, L’Aquila, Italy.

^e^ Microbiology Unit, Fondazione IRCCS Ca' Granda Ospedale Maggiore Policlinico, Milan, Italy.

**SUPPLEMENTAL MATERIAL**

**Table S1**. Compounds (n = 106) showing excellent activity (growth reduction ≥ 90% at spectrophotometric supernatant reading or CellTiter assay) against *P. aeruginosa* RP73 strain at the primary screening of the Drug Repurposing Compound Library (MedChem Express).

| Compound | CAS No. | Molecular weight | Target | Formula | Pathway | Research Area | Clinical Information |
| --- | --- | --- | --- | --- | --- | --- | --- |
| Finafloxacin | 209342-40-5 | 398.39 | Bacterial | C20H19FN4O4 | Anti-infection | Infection | Launched |
| Lomefloxacin (hydrochloride) | 98079-52-8 | 387.81 | Antibiotic; Bacterial | C17H20ClF2N3O3 | Anti-infection | Infection | Launched |
| Ciclopirox | 29342-05-0 | 207.27 | Autophagy; Bacterial; Ferroptosis; Fungal | C12H17NO2 | Anti-infection; Apoptosis; Autophagy | Infection; Cancer | Launched |
| Broxyquinoline | 521-74-4 | 302.95 | Parasite | C9H5Br2NO | Anti-infection | Infection | Launched |
| 3-AP | 143621-35-6 | 195.24 | DNA/RNA Synthesis | C7H9N5S | Cell Cycle/DNA Damage | Cancer | Phase 3 |
| Clioquinol | 130-26-7 | 305.50 | Antibiotic; Autophagy; Fungal; Mitophagy | C9H5ClINO | Anti-infection; Autophagy | Infection; Cancer | Launched |
| Ebselen | 60940-34-3 | 274.18 | Calcium Channel; HIV; Phosphatase; Virus Protease | C13H9NOSe | Anti-infection; Membrane Transporter/Ion Channel; Metabolic Enzyme/Protease; Neuronal Signaling | Cancer; Infection; Inflammation/Immunology; Neurological Disease | Phase 3 |
| Sulfisoxazole | 127-69-5 | 267.30 | Antibiotic; Bacterial; Endothelin Receptor | C11H13N3O3S | Anti-infection; GPCR/G Protein | Cancer; Infection; Endocrinology | Launched |
| Gepotidacin | 1075236-89-3 | 448.52 | Bacterial; Topoisomerase | C24H28N6O3 | Anti-infection; Cell Cycle/DNA Damage | Infection | Phase 3 |
| Sulfamonomethoxine | 1220-83-3 | 280.30 | Antibiotic; Bacterial | C11H12N4O3S | Anti-infection | Infection | Launched |
| Diiodohydroxyquinoline | 83-73-8 | 396.95 | Antibiotic; Bacterial | C9H5I2NO | Anti-infection | Infection | Launched |
| Cadazolid | 1025097-10-2 | 585.55 | Antibiotic; Bacterial | C29H29F2N3O8 | Anti-infection | Infection | Phase 3 |
| Delafloxacin (meglumine) | 352458-37-8 | 635.97 | Antibiotic; Bacterial | C25H29ClF3N5O9 | Anti-infection | Infection | Launched |
| Tebipenem | 161715-21-5 | 383.49 | Antibiotic; Bacterial | C16H21N3O4S2 | Anti-infection | Infection | Phase 3 |
| Thiamphenicol | 15318-45-3 | 356.22 | Antibiotic; Bacterial | C12H15Cl2NO5S | Anti-infection | Infection | Launched |
| Florfenicol | 73231-34-2 | 358.21 | Antibiotic; Bacterial | C12H14Cl2FNO4S | Anti-infection | Infection | Launched |
| Meclocycline (Sulfosalicylate Salt) | 73816-42-9 | 695.05 | Antibiotic; Bacterial | C29H27ClN2O14S | Anti-infection | Infection | Launched |
| Sulfadiazine | 68-35-9 | 250.28 | Antibiotic; Bacterial; Parasite | C10H10N4O2S | Anti-infection | Infection | Launched |
| SP-420 | 911714-45-9 | 355.41 | Others | C16H21NO6S | Others | Others | Phase 2 |
| Sulfamethoxazole | 723-46-6 | 253.28 | Antibiotic; Bacterial | C10H11N3O3S | Anti-infection | Infection | Launched |
| Faropenem daloxate | 141702-36-5 | 397.40 | Antibiotic; Bacterial | C17H19NO8S | Anti-infection | Infection | Launched |
| Prulifloxacin | 123447-62-1 | 461.46 | Antibiotic; Bacterial | C21H20FN3O6S | Anti-infection | Infection | Launched |
| Methacycline (hydrochloride) | 3963-95-9 | 478.88 | Antibiotic; Bacterial | C22H23ClN2O8 | Anti-infection | Infection | Launched |
| Levofloxacin (hydrate) | 138199-71-0 | 370.38 | Antibiotic; Bacterial | C18H20FN3O4 . 0.5H2O | Anti-infection | Infection | Launched |
| Cefepime (Dihydrochloride Monohydrate) | 123171-59-5 | 571.50 | Antibiotic; Bacterial | C19H28Cl2N6O6S2 | Anti-infection | Infection | Launched |
| Nitroxoline | 4008-48-4 | 190.16 | Antibiotic; Autophagy; Bacterial | C9H6N2O3 | Anti-infection; Autophagy | Infection; Cancer | Launched |
| Cefoperazone | 62893-19-0 | 645.67 | Antibiotic; Bacterial | C25H27N9O8S2 | Anti-infection | Infection | Launched |
| Piperacillin (sodium) | 59703-84-3 | 539.54 | Antibiotic; Bacterial | C23H26N5NaO7S | Anti-infection | Infection | Launched |
| Epetraborole (hydrochloride) | 1234563-16-6 | 273.52 | Bacterial | C11H17BClNO4 | Anti-infection | Infection | Phase 2 |
| Zinc Pyrithione | 13463-41-7 | 317.69 | Bacterial; Fungal; Proton Pump | C10H8N2O2S2Zn | Anti-infection; Membrane Transporter/Ion Channel | Cardiovascular Disease | Launched |
| Piroctone olamine | 68890-66-4 | 298.42 | Fungal | C16H30N2O3 | Anti-infection | Infection | Launched |
| Tavaborole | 174671-46-6 | 151.93 | Antibiotic; Fungal | C7H6BFO2 | Anti-infection | Infection | Launched |
| Resveratrol | 501-36-0 | 228.24 | Antibiotic; Apoptosis; Autophagy; Bacterial; Fungal; IKK; Keap1-Nrf2; Mitophagy; Sirtuin | C14H12O3 | Anti-infection; Apoptosis; Autophagy; Cell Cycle/DNA Damage; Epigenetics; NF-κB | Cancer; Infection; Inflammation/Immunology | Launched |
| Nalidixic acid | 389-08-2 | 232.24 | Antibiotic; Bacterial; Topoisomerase | C12H12N2O3 | Anti-infection; Cell Cycle/DNA Damage | Infection | Launched |
| Sultamicillin (tosylate) | 83105-70-8 | 766.86 | Bacterial | C32H38N4O12S3 | Anti-infection | Infection | Launched |
| Chlortetracycline (hydrochloride) | 64-72-2 | 515.34 | Antibiotic; Bacterial | C22H24Cl2N2O8 | Anti-infection | Infection | Launched |
| Panobinostat | 404950-80-7 | 349.43 | Apoptosis; Autophagy; HDAC; HIV | C21H23N3O2 | Anti-infection; Apoptosis; Autophagy; Cell Cycle/DNA Damage; Epigenetics | Cancer | Launched |
| Doripenem (monohydrate) | 364622-82-2 | 438.52 | Antibiotic; Bacterial | C15H26N4O7S2 | Anti-infection | Infection | Launched |
| Pazufloxacin (mesylate) | 163680-77-1 | 414.41 | Antibiotic; Bacterial | C17H19FN2O7S | Anti-infection | Infection | Launched |
| Carmofur | 61422-45-5 | 257.26 | Nucleoside Antimetabolite/Analog; SARS-CoV; Virus Protease | C11H16FN3O3 | Anti-infection; Cell Cycle/DNA Damage | Cancer | Launched |
| Aztreonam | 78110-38-0 | 435.43 | Antibiotic; Bacterial | C13H17N5O8S2 | Anti-infection | Infection | Launched |
| Tobramycin | 32986-56-4 | 467.51 | Antibiotic; Bacterial | C18H37N5O9 | Anti-infection | Infection | Launched |
| Trovafloxacin | 147059-72-1 | 416.35 | Antibiotic; Bacterial; Topoisomerase | C20H15F3N4O3 | Anti-infection; Cell Cycle/DNA Damage | Infection | Launched |
| Auranofin | 34031-32-8 | 680.50 | Bacterial; SARS-CoV | C20H36AuO9PS | Anti-infection | Cancer; Infection; Inflammation/Immunology | Launched |
| Novobiocin (Sodium) | 1476-53-5 | 634.61 | Antibiotic; Autophagy; Bacterial | C31H35N2NaO11 | Anti-infection; Autophagy | Infection; Cancer | Launched |
| Cinoxacin | 28657-80-9 | 262.22 | Antibiotic; Bacterial | C12H10N2O5 | Anti-infection | Infection | Launched |
| Bronopol | 52-51-7 | 199.99 | Bacterial | C3H6BrNO4 | Anti-infection | Infection | Launched |
| Zoliflodacin | 1620458-09-4 | 487.44 | Bacterial; DNA/RNA Synthesis | C22H22FN5O7 | Anti-infection; Cell Cycle/DNA Damage | Infection | Phase 3 |
| Nadifloxacin | 124858-35-1 | 360.38 | Antibiotic; Bacterial | C19H21FN2O4 | Anti-infection | Infection | Launched |
| Ozenoxacin | 245765-41-7 | 363.41 | Bacterial | C21H21N3O3 | Anti-infection | Infection | Launched |
| Faropenem sodium | 122547-49-3 | 307.30 | Antibiotic; Bacterial | C12H14NNaO5S | Anti-infection | Infection | Launched |
| Oxytetracycline | 79-57-2 | 460.43 | Antibiotic; Bacterial; Endogenous Metabolite; HSV | C22H24N2O9 | Anti-infection; Metabolic Enzyme/Protease | Infection | Launched |
| Gatifloxacin (hydrochloride) | 121577-32-0 | 411.86 | Antibiotic; Bacterial; Topoisomerase | C19H23ClFN3O4 | Anti-infection; Cell Cycle/DNA Damage | Infection | Launched |
| Oxytetracycline (hydrochloride) | 2058-46-0 | 496.89 | Antibiotic; Bacterial; Endogenous Metabolite; HSV | C22H25ClN2O9 | Anti-infection; Metabolic Enzyme/Protease | Infection | Launched |
| Norfloxacin | 70458-96-7 | 319.33 | Antibiotic; Bacterial; Endogenous Metabolite | C16H18FN3O3 | Anti-infection; Metabolic Enzyme/Protease | Infection | Launched |
| 5-Fluorouracil | 51-21-8 | 130.08 | Apoptosis; Endogenous Metabolite; HIV; Nucleoside Antimetabolite/Analog | C4H3FN2O2 | Anti-infection; Apoptosis; Cell Cycle/DNA Damage; Metabolic Enzyme/Protease | Cancer | Launched |
| Sulforaphane | 4478-93-7 | 177.29 | Apoptosis; HDAC; Keap1-Nrf2 | C6H11NOS2 | Apoptosis; Cell Cycle/DNA Damage; Epigenetics; NF-κB | Cancer; Inflammation/Immunology | Phase 3 |
| Tetracycline (hydrochloride) | 64-75-5 | 480.90 | Antibiotic; Bacterial | C22H25ClN2O8 | Anti-infection | Infection | Launched |
| Silver sulfadiazine | 22199-08-2 | 357.14 | Antibiotic; Bacterial; DNA/RNA Synthesis | C10H9AgN4O2S | Anti-infection; Cell Cycle/DNA Damage | Infection | Launched |
| Sisomicin (sulfate) | 53179-09-2 | 692.72 | Antibiotic; Bacterial | C19H37N5O7 . 5/2 H2SO4 | Anti-infection | Infection | Launched |
| Sitafloxacin (hydrate) | 163253-35-8 | 436.84 | Antibiotic; Bacterial | C19H18ClF2N3O3.3/2H2O | Anti-infection | Infection | Launched |
| Danofloxacin (mesylate) | 119478-55-6 | 453.48 | Antibiotic; Bacterial | C20H24FN3O6S | Anti-infection | Infection | Launched |
| Gatifloxacin | 112811-59-3 | 375.39 | Antibiotic; Bacterial; Topoisomerase | C19H22FN3O4 | Anti-infection; Cell Cycle/DNA Damage | Infection | Launched |
| Eravacycline (dihydrochloride) | 1334714-66-7 | 631.48 | Bacterial | C27H33Cl2FN4O8 | Anti-infection | Infection | Launched |
| Rifamycin (sodium) | 14897-39-3 | 719.75 | Antibiotic; Bacterial | C37H46NNaO12 | Anti-infection | Infection | Launched |
| Tolcapone | 134308-13-7 | 273.24 | Amyloid-β; COMT | C14H11NO5 | Metabolic Enzyme/Protease; Neuronal Signaling | Neurological Disease | Launched |
| Rifapentine | 61379-65-5 | 877.03 | Antibiotic; Bacterial | C47H64N4O12 | Anti-infection | Infection | Launched |
| Sulfacetamide | 144-80-9 | 214.24 | Antibiotic; Bacterial | C8H10N2O3S | Anti-infection | Infection | Launched |
| Sulfaphenazole | 526-08-9 | 314.36 | Bacterial | C15H14N4O2S | Anti-infection | Infection | Launched |
| Sulfabenzamide | 127-71-9 | 276.31 | Antibiotic; Autophagy; Bacterial | C13H12N2O3S | Anti-infection; Autophagy | Infection | Launched |
| Furagin | 1672-88-4 | 264.19 | Bacterial | C10H8N4O5 | Anti-infection | Infection | Launched |
| Mezlocillin (sodium) | 42057-22-7 | 561.56 | Antibiotic; Bacterial | C21H24N5NaO8S2 | Anti-infection | Infection | Launched |
| XL-784 | 1224964-36-6 | 548.92 | MMP | C21H21ClF2N3O8S- | Metabolic Enzyme/Protease | Cardiovascular Disease | Phase 2 |
| Tocofersolan | 9002-96-4 | 1513.00 | Others | N/A | Others | Metabolic Disease | Launched |
| Tirapazamine | 27314-97-2 | 178.15 | Others | C7H6N4O2 | Others | Cancer | Phase 3 |
| RRx-001 | 925206-65-1 | 268.02 | Apoptosis; Parasite | C5H6BrN3O5 | Anti-infection; Apoptosis | Cancer; Infection; Inflammation/Immunology | Phase 3 |
| Radafaxine (hydrochloride) | 106083-71-0 | 292.20 | Monoamine Transporter | C13H19Cl2NO2 | Membrane Transporter/Ion Channel | Neurological Disease | Phase 2 |
| Ofloxacin | 82419-36-1 | 361.37 | Antibiotic; Bacterial | C18H20FN3O4 | Anti-infection | Infection | Launched |
| Rifampicin | 13292-46-1 | 822.94 | Antibiotic; Bacterial; Influenza Virus | C43H58N4O12 | Anti-infection | Infection | Launched |
| Colistin (sulfate) | 1264-72-8 | 1253.51 | Antibiotic; Autophagy; Bacterial | C52H100N16O17S | Anti-infection; Autophagy | Infection | Launched |
| Rifamycin S | 13553-79-2 | 695.75 | Antibiotic; Bacterial; Reactive Oxygen Species | C37H45NO12 | Anti-infection; Immunology/Inflammation; Metabolic Enzyme/Protease; NF-κB | Infection | Phase 3 |
| Fosfomycin (sodium) | 26016-99-9 | 182.02 | Antibiotic; Bacterial | C3H5Na2O4P | Anti-infection | Infection | Launched |
| Furazolidone | 67-45-8 | 225.16 | Antibiotic; Apoptosis; Bacterial | C8H7N3O5 | Anti-infection; Apoptosis | Infection | Launched |
| Sulfadimethoxine (sodium) | 1037-50-9 | 332.31 | Antibiotic; Bacterial | C12H13N4NaO4S | Anti-infection | Infection | Launched |
| Sulbactam | 68373-14-8 | 233.24 | Antibiotic; Bacterial | C8H11NO5S | Anti-infection | Infection | Launched |
| L-SelenoMethionine | 3211-76-5 | 196.11 | Apoptosis; Endogenous Metabolite | C5H11NO2Se | Apoptosis; Metabolic Enzyme/Protease | Cancer | Launched |
| Merbromin | 129-16-8 | 752.67 | Bacterial | C20H10Br2HgNa2O6 | Anti-infection | Others | Launched |
| Sparfloxacin | 110871-86-8 | 392.40 | Antibiotic; Bacterial | C19H22F2N4O3 | Anti-infection | Infection | Launched |
| Enoxacin (hydrate) | 84294-96-2 | 347.34 | Antibiotic; Bacterial; DNA/RNA Synthesis; MicroRNA | C15H17FN4O3 . 3/2 H2O | Anti-infection; Cell Cycle/DNA Damage; Epigenetics | Infection; Cancer | Launched |
| Rifaximin | 80621-81-4 | 785.88 | Antibiotic; Bacterial | C43H51N3O11 | Anti-infection | Infection | Launched |
| Bithionol | 97-18-7 | 356.05 | Parasite | C12H6Cl4O2S | Anti-infection | Cancer | Launched |
| Garenoxacin (Mesylate hydrate) | 223652-90-2 | 540.53 | Bacterial | C24H26F2N2O8S | Anti-infection | Infection | Launched |
| Cefoperazone (sodium salt) | 62893-20-3 | 667.65 | Antibiotic; Bacterial | C25H26N9NaO8S2 | Anti-infection | Infection | Launched |
| Ribavirin | 36791-04-5 | 244.20 | Antibiotic; HCV; RSV | C8H12N4O5 | Anti-infection | Infection | Launched |
| Pefloxacin (mesylate) | 70458-95-6 | 429.46 | Antibiotic; Bacterial | C18H24FN3O6S | Anti-infection | Infection | Launched |
| Cefozopran (hydrochloride) | 113981-44-5 | 551.99 | Antibiotic; Bacterial | C19H18ClN9O5S2 | Anti-infection | Infection | Launched |
| Levofloxacin | 100986-85-4 | 361.37 | Antibiotic; Bacterial | C18H20FN3O4 | Anti-infection | Infection | Launched |
| Doxycycline (hyclate) | 24390-14-5 | 512.94 | Antibiotic; Bacterial; MMP | C22H24N2O8.1/2C2H6O.ClH.1/2H2O | Anti-infection; Metabolic Enzyme/Protease | Infection | Launched |
| Demeclocycline (hydrochloride) | 64-73-3 | 501.31 | Antibiotic; Bacterial | C21H22Cl2N2O8 | Anti-infection | Infection | Launched |
| Chlorquinaldol | 72-80-0 | 228.07 | Antibiotic; Bacterial; Fungal | C10H7Cl2NO | Anti-infection | Infection | Launched |
| Gemifloxacin (mesylate) | 210353-53-0 | 485.49 | Bacterial | C19H24FN5O7S | Anti-infection | Infection | Launched |
| Sulfachloropyridazine | 80-32-0 | 284.72 | Antibiotic; Bacterial | C10H9ClN4O2S | Anti-infection | Infection | Launched |
| Sulfadiazine (sodium) | 547-32-0 | 272.26 | Antibiotic; Bacterial; Parasite | C10H9N4NaO2S | Anti-infection | Infection | Launched |
| Tosufloxacin (tosylate hydrate) | 1400591-39-0 | 594.56 | Antibiotic; Bacterial | C26H25F3N4O7S | Anti-infection | Infection | Launched |
| Fidaxomicin | 873857-62-6 | 1058.04 | Antibiotic; Apoptosis; Bacterial | C52H74Cl2O18 | Anti-infection; Apoptosis | Infection | Launched |
| Ciprofloxacin | 85721-33-1 | 331.34 | Antibiotic; Bacterial | C17H18FN3O3 | Anti-infection | Infection | Launched |

**Table S2**. Genotypic and phenotypic traits of the selected panel of *P. aeruginosa* CF strains.

| Strain ID | MLST | Serotype | Infection status | Mucoid phenotype | Tobramycin resistance | MDR^a^ | Resistance genes | Biofilm^d^ | Pyocyanin^e^ | Pyoverdine^f^ | Protease^g^ |
| --- | --- | --- | --- | --- | --- | --- | --- | --- | --- | --- | --- |
| FM3986 | ST393 | O6 | chronic | + | - | - | *aph(3’)-IIb, bla_OXA-50_, bla_PAO_, catB7, fosA* | 1.969 | 3.28 | 16.49 | 10.7 |
| RI3988 | ST252 | O1 | chronic | - | - | - | *aph(3’)-IIb, bla_OXA-50_, bla_PAO_, catB7, fosA* | 2.258 | 4.18 | 6.11 | NP^b^ |
| GR3957 | ST446 | O11 | sporadic | - | - | - | *aph(3’)-IIb, bla_OXA-50_, catB7* | 1.297 | 3.79 | 16.88 | NP |
| BJ3525 | New | O1 | first | - | - | - | *aph(3’)-IIb, bla_OXA-50_, bla_PAO_, catB7, fosA* | 0.644 | 6.76 | 19.76 | 12.5 |
| MG3871 | New | O1 | chronic | - | + | + | *aph(3’)-IIb, bla_OXA-50_, bla_PAO_, catB7, fosA* | 1.875 | 4.96 | 11.36 | 15.8 |
| CA3983 | ST 1094 | O9 | chronic | - | + | + | *aph(3’)-IIb, bla_OXA-50_, bla_PAO_, catB7* | 2.534 | 1.04 | 4.40 | NP |
| SM3483 | New | O11 | chronic | - | + | + | *aph(3’)-IIb, bla_OXA-50_, bla_PAO_, catB7, fosA* | 1.659 | NT^c^ | NT | NT |
| BA3671 | ST555 | O6 | first | - | - | - | *bla_OXA-50_, catB7, fosA* | 1.102 | 9,4 | 31.2 | 21 |
| NF3975 | ST1605 | O9 | first | - | - | - | *aph(3’)-IIb, bla_OXA-50_, catB7, fosA* | 1.147 | 4,2 | 13.9 | 30 |
| VA3949 | New | O5 | chronic | - | + | + | *aph(3’)-IIb, bla_OXA-50_, catB7* | 1.617 | 0.37 | 1.9 | NP |

^a^ MDR, multidrug-resistant strain, if non-susceptible to at least one agent in three or more antimicrobial categories among those tested (β-lactam/ β-lactamase inhibitor, aminoglycosides, fluoroquinolones, phosphonic acid derivative, polymyxin, carbapenems, and cephalosporins) [1].

^b^ NP, not a producer.

^c^ NT, not tested.

^d^ Biofilm biomass formed after 48 h-incubation under “CF-like” conditions; values are expressed as OD_492_. All strains could be classified as strong biofilm producers, according to Stepanovic et al. [2].

^e^ Values are expressed as OD_520_ x 10^-2^.

^f^ Values are expressed as OD_400_ x 10^-2^.

^g^ Values are expressed as OD_600_ x 10^-2^.

**Table S3.** Virulence traits of the selected *P. aeruginosa* strains as assessed by whole-genome sequencing.

| STRAIN | VIRULENCE FACTORS | | | | | | | | | | |
| --- | --- | --- | --- | --- | --- | --- | --- | --- | --- | --- | --- |
|  | **Adherence** | **Antimicrobial activity** | **Antiphagocytosis** | **Biosurfactant** | **Enzyme** | **Iron uptake** | **Protease** | **Quorum sensing** | **Regulation** | **Secretion system** | **Toxin** |
| FM 3986 | *flaG*  *fleN, Q, R, S*  *flgA, B, C, D, E, F, G, H, I, J, K, L, M, N*  *flhA*  *fliA, C, D, E, F, G, H, I, J, K, L, M, N, Q, R, S, T*  *motA, B, C, D, Y*  *fimT, U, V*  *pilB, C, D, E, F, G, H, I, J, M, N, O, P, Q, R, S, T, U, V, W, X, Y2, Z*  *chpA, B, C, D, E* | *phzC1, D1, G1, M* | *alg44, 8, A, C, D, E, F, G, I, J, K, L, Q, R, U, W, X, Z*  *mucA, B, C, D, E, P* | *rhlA-genome, B, C* | *plcH, N, B* | *fptA*  *pchA, B, C, D, E, H, I, R*  *fpvA*  *pvdA, E, F, H, N, O, P, Q, S, Y* | *aprA*  *lasA, B*  *prpL* | *hdtS*  *lasI, R* | *gacA* | *clpV1*  *hcp1*  *icmF1*  *ppkA*  *pppA*  *vgrG1*  *exoS, T, Y*  *exsA, B, C, D*  *pcr1, 2, 3, 4, D, G, H, R, V*  *popB, D, N*  *pscB, C, F, G, I, J, L, N, P, Q, R, S, T, U* | *toxA*  *hcnA, C* |
| RI 3988 | *fleN, Q, R, S*  *flgB, C, D, F, G, H, J, M*  *flhF*  *fliA, E, F, G, H, J, L, M, N, O, P, Q, R, S*  *motA, B, C, D, Y*  *fimT*  *pilB, C, F, G, H, J, K, M, N, O, P, Q, R, S, U, W, Y2, Z*  *chpE* | *phzD1, E1, H, M* | *alg8, C, D, E, G, I, L, X, Z*  *mucA, B, D, E, P* | *rhlA, B, R* | *plcH, N* | *fptA*  *pchB, C, D, I, R*  *fpvA*  *pvdD, E, F, G, L, M, N, P, Q, S* | *aprA*  *lasA, B*  *prpL* | *hdtS*  *lasI, R* | *gacA, S* | *fha1*  *vgrG1*  *exoS, T*  *exsA, D, E*  *pcr1, 2, 3, D*  *popD, N*  *pscF, H, I, J, L, N, R, T, U* | *toxA*  *hcnA* |
| GR 3957 | *flgL*  *flhA, F*  *fliP*  *pilB, Q, S*  *chpA, E* |  | *alg8, A, I, J, Z*  *mucP* | *rhlB, I* | *plcH* | *fptA*  *pvdA, D, E, I, J* | *lasA, B*  *prpL* |  |  | *icmF1*  *exoT*  *pcrR*  *pscD, J* | *hcnB* |
| BJ 3525 | *flaG*  *fleN, Q, R, S*  *flgA, B, C, D, E, F, G, H, I, J, K, L, M, N*  *flhA, B, F*  *fliA, C, D, E, F, G, H, I, J, K, L, M, N, P, Q, R, S, T*  *motA, B, C, Y*  *fimT, U, V*  *pilB, C, D, E, G, H, J, K, M, N, O, P, Q, R, S, T, U, V, W, X, Y1, Y2, Z*  *chpA, B, D, E* | *phzC1, D1, E1, F1, H, M, S* | *alg44, 8, A, C, D, E, G, I, J, K, L, Q, U, W, Z*  *mucB, C, D, E, P* | *rhlA, B, C* | *plcH, N, B*  *pldA* | *fptA*  *pchA, B, C, D, E, F, G, H, I, R*  *fpvA*  *pvdA, D, E, F, G, H, M, N, O, P, Q, S, Y* | *aprA*  *lasA, B*  *prpL* | *hdtS*  *rhlI, R*  *lasI, R* | *gacA, S* | *clpV1*  *fha1*  *icmF1*  *ppkA*  *pppA*  *vgrG1*  *exoT, Y*  *exsA, B, C, D, E*  *pcr1, 2, 3, 4, D,*  *G, H, R, V*  *popB, D, N*  *pscB, C, D, F, G, H, I, J, L, N, P, Q, R, S, T, U* | *toxA*  *hcnA, B, C* |
| MG 3871 | *flaG*  *fleN*  *flgA, B, C, D, E, F, G, H, I, J, K, L, M, N*  *flhA, B, F*  *fliA, C, D, E, F, H, I, J, K, L, M, P, Q, R, S, T*  *motA, C, D, Y*  *fimT, U, V*  *pilB, C, D, F, G, H, J, K, M, N, O, P, Q, R, S, T, U, V, W, X, Y1, Y2, Z*  *chpA, B, C, E* | *phzD1, E1, F1, G2, M, S* | *alg44, 8, A, C, D, E, G, J, K, L, Q, U, W, X, Z*  *mucA, B, D, E, P* | *rhlA, B, C* | *plcH, B* | *pchA, B, fptA*  *C, D, E, F, H, I, R*  *pldA*  *fpvA*  *pvdA, D, E, F, G, H, I, J, L, M, N, O, P, Q, S* | *aprA*  *lasA, B*  *prpL* | *lasI, R*  *hdtS*  *rhlI, R* | *gacA, S* | *clpV1*  *fha1*  *icmF1*  *hcp1*  *ppkA*  *pppA*  *vgrG1*  *exoT, U, Y*  *exsA, B, C, D*  *pcr1, 2, 3, 4, D, G, H, R, V*  *popB, D, N*  *pscC, D, F, G, H, I, J, K, L, N, P, Q, S, T, U* | *toxA*  *hcnA, B, C* |
| CA 3983 | *fleN, Q, R, S*  *flgA, B, C, D, E, F, G, H, I, J, K, L, M, N*  *flhA, B, F*  *fliE, F, G, H, J, K, L, M, N, P, Q, R, S, T*  *motA, B, C, D, Y*  *fimT, U, V*  *pilA, B, C, D, E, G, H, J, K, M, N, O, P, Q, R, S, T, U, V, W, X, Y1, Y2, Z*  *chpA, B, C, E* | *phzB1, B2, C1, D1, E1, F1, M, S* | *alg44, 8, A, C, D, E, G, I, J, K, L, Q, U, W, X, Z*  *mucB, C, D, E, P* | *rhlA, B, C* | *plcH, N, B* | *pchA, B, C, D, E, F, H, I, R*  *fpvA*  *pvdA, D, E, F, G, H, I, M, N, O, P, Q, S, Y* | *aprA*  *lasA, B*  *prpL* | *lasI, R*  *rhlI, R* | *gacA, S* | *clpV1*  *hcp1*  *ppkA*  *pppA*  *vgrG1*  *exoS, T, Y*  *exsA, C, D*  *pcr1, 2, 3, 4, D, G, H, R, V*  *popB, D, N*  *pscB, C, D, F, G, H, I, J, L, N, P, Q, R, S, T, U* | *toxA*  *hcnA, C* |
| SM 3483 | *flaG*  *fleN, Q, R, S*  *flgA, B, C, D, E, F, G, H, I, J, K, L, M, N*  *flhA, B, F*  *fliA, C, D, E, F, G, H, I, J, K, L, M, N, P, Q, R, S, T*  *motA, B, C, Y*  *fimT, U, V*  *pilA, B, C, D, E, G, H, J, K, M, N, O, P, Q, R, S, T, U, V, W, X, Y1, Y2, Z*  *chpA, B, C, E* | *phzB1, B2, C1, D1, E1, F1, M, S* | *alg44, 8, A, C, D, E, G, I, J, K, L, Q, U, W, Z*  *mucB, C, D, E, P* | *rhlA, B, C* | *plcH, N, B* | *pchA, B, C, D, E, F, H, I, R*  *fpvA*  *pvdA, D, E, F, G, H, I, M, N, O, P, Q, S, Y* | *aprA*  *lasA, B*  *prpL* | *lasI, R*  *rhlI, R* | *gacA, S* | *clpV1*  *hcp1*  *ppkA*  *pppA*  *vgrG1*  *exoS, T, Y*  *exsA, C, D*  *pcr1, 2, 3, 4, D, G, H, R, V*  *popB, D, N*  *pscB, C, D, F, G, H, I, J, L, N, P, Q, R, S, T, U* | *toxA*  *hcnA, C* |
| BA 3671 | *flgE, M*  *flhA*  *fliG, K, L*  *pilB, N, Q, Y2, H* | *phzC1, H, S* | *alg8, A, C, D, K, Z*  *mucD, P* | *rhlC* | *plcH* | *fptA*  *pvdE, I, Q* | *lasB* |  | *gacS* | *icmF1*  *ppkA*  *vgrG1*  *exoS, Y*  *popB, D*  *pscD, F, I* | *toxA* |
| NF 3975 | *fimV*  *pilY1*  *chpA* | *phzE1* | *algE, I* |  |  | *pchI*  *fpvA*  *pvdE, I, M, N, P, Q* | *aprA*  *lasA* |  |  | *clpV1*  *exoY*  *pcrD, R, V*  *pscC, K, R* |  |
| VA 3949 | *flaG*  *fleN, R, S*  *flgB, C, D, F, I, J, K, L, M, N*  *flhA*  *fliA, C, D, E, F, G, J, K, L, M, P, Q, R, S, T*  *motA, B, Y*  *fimU*  *pilB, D, E, F, G, H, M, O, P, Q, R, S, U, V, X, Y1, Y2, Z*  *chpA, C, D, E* | *phzD1, E1, F1, G1, H, S* | *alg44, 8, A, C, D, E, G, I, J, K, L, Q, R, U, W, X, Z*  *mucA, D* | *rhlC* | *plcH* | *pchB, C, D, E, F, I, R*  *fpvA*  *pvdA, E, F, G, H, I, M, N, O, P, Q* | *lasA, B*  *prpL* | *lasI*  *rhlR* | *gacA, S* | *clpV1*  *hcp1*  *pppA*  *vgrG1*  *exoY*  *exsA, D*  *pcr1, 2, 4, D, H, V*  *popB, D*  *pscD, F, H, I, J, L, R, S, T, U* | *hcnB, C* |

**FIG S1** *In vitro* cytotoxicity against IB3-1 cells. Cell monolayers were exposed for 24 h to different concentrations of each hit, and the cell viability was assessed using a tetrazolium-based colorimetric assay. Control samples were not exposed. Results are shown as mean OD_492_ + SD (n = 6). Significant differences at Ordinary one-way ANOVA corrected for multiple comparisons by Holm-Sidak post-hoc test: * p<0.05, ** *p*<0.01, and *** *p*<0.001, and **** *p*<0.0001 *vs*. control.

**REFERENCES**

1. Magiorakos AP, Srinivasan A, Carey RB, Carmeli Y, Falagas ME, Giske CG, Harbarth S, Hindler JF, Kahlmeter G, Olsson-Liljequist B, Paterson DL, Rice LB, Stelling J, Struelens MJ, Vatopoulos A, Weber JT, Monnet DL. 2012. Multidrug-resistant, extensively drug-resistant and pandrug-resistant bacteria: an international expert proposal for interim standard definitions for acquired resistance. *Clin Microbiol Infect* 18(3):268-81.

2. Stepanović S, Vuković D, Hola V, Di Bonaventura G, Djukić S, Cirković I, Ruzicka F. 2007. Quantification of biofilm in microtiter plates: overview of testing conditions and practical recommendations for assessment of biofilm production by staphylococci. *APMIS* 115(8):891-9.
